# “A real eye opener” – development, implementation and evaluation of a seminar on interprofessional teamwork in GP specialty training

**DOI:** 10.3205/zma001488

**Published:** 2021-06-15

**Authors:** Katharina Dippell, Maria-Sophie Brueckle, Monika Sennekamp, Armin Wunder, Lia Pauscher, Pamela Reißner, Ferdinand Gerlach, Karola Mergenthal

**Affiliations:** 1 Goethe Universität, Institut für Allgemeinmedizin, Frankfurt/Main, Germany

**Keywords:** interprofessional collaboration, interprofessional team, team communication, primary care

## Abstract

**Introduction: **The successful treatment of a multitude of chronic diseases is largely dependent on effective interprofessional collaboration. In Germany, many healthcare employees feel unprepared for the challenges of interprofessional teamwork. Can a seminar designed by an interprofessional team for an interprofessional target group improve mutual understanding and provide the basis for successful interprofessional collaboration?

**Methodological approach: **For the development of the seminar, Kern’s model for curriculum development was used, which includes the following six steps: problem identification, needs analysis, goal definition (learning objectives and learning content), educational strategies, implementation and evaluation. The all-day event brought together physiotherapists, pharmacists, medical assistants and doctors undergoing specialist training in family medicine. Representatives of the various professions were recruited through multiple channels (associations, pharmacist societies, alumni-networks, etc.). Practical examples and role-play were used to develop a better understanding of each other’s professions and to support goal-oriented and appreciative communication among them. The seminar belongs to the curriculum provided by the Hesse Competence Center for Specialist Training in General Practice and has so far taken place three times. The evaluation was carried out by means of a detailed, self-designed questionnaire with five open and 20 closed questions. The statistical analysis was mainly descriptive (mean value, minimum, maximum and SD).

**Results: **Overall, 29 persons have participated in the workshops (eleven doctors undergoing specialist training, six physiotherapists, six pharmacists, six medical assistants). Overall, the seminar was rated very highly. Individual aspects of the seminar’s design, such as relevance and practice-orientation, as well as an assessment of whether the seminar contributed towards increasing participants’ ability to collaborate with other professionals, were rated positively. In addition, a pre-post comparison of self-assessed ability to collaborate interprofessionally showed significant improvement.

**Discussion: **This highly interactive one-day seminar design contributed towards improving understanding for other professions and communication skills. In order to achieve a broad-based improvement in interprofessional collaboration over the long term, this or similar concepts should be employed more extensively.

**Conclusions: **The results suggest that participants in interprofessional seminars consider them to improve interprofessional collaboration.

## Zusammenfassung

**Einleitung:** Bei vielen chronischen Erkrankungen hängt der Behandlungserfolg auch von gelungener interprofessioneller Zusammenarbeit ab. In Deutschland fühlen sich viele Beschäftigte im Gesundheitswesen nicht auf die Herausforderungen interprofessioneller Teamarbeit vorbereitet. Kann ein im interprofessionellen Team konzipiertes Seminar mit einer interprofessionell zusammengesetzten Zielgruppe das Verständnis der Professionen füreinander verbessern und damit der Grundstein für eine erfolgreiche interprofessionelle Zusammenarbeit sein?

**Methodische Vorgehensweise: **Für die Entwicklung des Seminars wurde das Konzept des Kern-Zyklus zu Hilfe genommen, welches die sechs Schritte der Problemidentifizierung, speziellen Bedarfsanalyse, Zieldefinition (Lernziele und Lerninhalte), didaktische Umsetzung, Implementierung und Evaluation enthielt. Das ganztägige Seminar bringt Physiotherapeut*innenn, Apotheker*innen, VERAH und ÄiW zusammen. Die Professionen wurden über unterschiedliche Wege (Verbände, Apothekerkammer, Alumni-Vernetzung etc.) angeworben. Mit praktischen Beispielen und Rollenspielen wird ein besseres Verständnis der jeweils anderen Professionen und Hilfestellungen zu zielführender und gleichzeitig wertschätzender Kommunikation miteinander erarbeitet. Es findet im Rahmen des Seminarprogramms des Kompetenzzentrums Weiterbildung Hessen statt und wurde bisher dreimal angeboten. Die Evaluation erfolgte mittels eines selbstkonzipierten, ausführlichen Fragenbogens mit fünf offenen und 20 geschlossenen Fragen. Die statistische Analyse erfolgte überwiegend deskriptiv (Mittelwert, Minimum, Maximum und SD).

**Ergebnisse: **Insgesamt 29 Personen nahmen an den Seminaren teil, elf ÄiW, sechs Physiotherapeuten/innen, sechs Apotheker/innen, sechs Medizinische Fachangestellte (MFA). Insgesamt wurden die Seminare sehr gut bewertet. Die einzelnen Aspekte der Seminargestaltung wie die Relevanz und Praxisnähe sowie die Beurteilung, ob das Seminar dazu beiträgt, die eigene Kompetenz in der Zusammenarbeit mit anderen Professionen zu steigern, wurden positiv bewertet. Zudem zeigte sich im Prä-Post-Vergleich der Selbsteinschätzung bezüglich der eigenen Fähigkeit zur interprofessionellen Zusammenarbeit eine signifikante Verbesserung.

**Diskussion: **Dieses sehr interaktive eintägige Seminarkonzept hat dazu beigetragen, dass das Verständnis für die anderen Professionen und die Kommunikationsfähigkeit sich verbessert haben. Damit langfristig und breitflächig eine Verbesserung in der interprofessionellen Zusammenarbeit stattfinden kann, müssen dieses oder ähnliche Konzepte ausgeweitet werden.

**Schlussfolgerungen: **Die Ergebnisse deuten darauf hin, dass interprofessionelle Seminare in der Selbsteinschätzung zu einer besseren interprofessionellen Zusammenarbeit beitragen können.

## 1. Introduction

As a result of demographic change and an associated increase in the overall number of multimorbid patients, successful interprofessional cooperation is being increasingly highlighted and called for (e.g. by the German Advisory Council on the Assessment of Developments in the Health Care System (SVR) 2014 [1] and the WHO [2]. In Europe, over 50 million persons are currently multimorbid and this number will continue to rise in the future [3]. As treatment success for many diseases is dependent on interprofessional collaboration, several guidelines have emphasized its importance [4], [5]. 

Patients, family practitioners, and other healthcare professionals, are faced with the challenges involved in the provision of complex healthcare services for multimorbidity and polypharmacy on a daily basis [6]. These patients and healthcare professionals with a wide range of qualifications (nursing and therapeutic professions, physicians, pharmacists, health care assistants, social workers etc.), and other involved persons (helpers in the home, caregiving relatives etc.), all participate in the implementation of treatment strategies. This collaboration should ideally be harmonized on all levels, with the ultimate aim of providing patient-centered treatment.

The need for efficient, effective and economically viable personnel deployment has increasingly provided federal states with an argument to support interprofessional collaboration [7]. However, many healthcare employees in Germany do not feel they have been prepared to meet the challenges of working on an interprofessional team [1]. Studies show that a lack of information and misunderstandings are responsible for delaying and even endangering the success of treatment. As a result, not only do around 80% of all serious mistakes in treatment procedures stem from communication errors, but most preventable adverse events are also the result of inadequate communication. A shared language for the transfer of information would support error-free communication [8]. It is therefore hardly surprising that the need for successful interprofessional communication and the ability to work in an interprofessional team are increasingly part of medical education and specialty training. Throughout Germany, numerous trainings and study modules have begun to include interdisciplinary and interprofessional learning content in their programs [9], [10], [11]. In addition, the recently approved Masterplan 2020 for the reform of medical studies foresees the inclusion of interprofessional course content [12]. In this connection, the German medical journal Deutsche Ärzteblatt (2017) [13], has written: “Coordinated interaction will be more common and more effective, the earlier it is, for example, practiced and becomes used in medical training.” At the Competence Center for Specialist Training, we have close contact to physicians undergoing specialty training, and we plan to exploit this to strengthen interprofessional collaboration in day-to-day working life. This has enabled us to take on board the learning principle that content that is applied by trainees in role play or real situations, is more easily absorbed and translated into ready-to-use, integrated and consistent knowledge. As a result, it is easier to recall and make use of such knowledge in concrete situations [14].

In the working group responsible for specialty training, the idea arose that colleagues from differing professions at the Institute of General Practice in Frankfurt am Main should develop, implement and evaluate an interprofessional seminar for physicians undergoing such training. The seminar was included in the Hesse Competence Center for Specialist Training’s education program, which was established as part of the “Hesse pact to safeguard the provision of healthcare” in 2012. 

The present paper describes the development, content and evaluation of the continuing seminar. When developing the content of the seminar, the following research question was posed: 

Can a seminar that is developed by an interprofessional team for an interprofessional target group achieve the following goals with the help of practical examples and role play:

- more intensive preparation for interprofessional collaboration,
- improvement in communication with other professional groups,
- improved comprehension of the particular competencies of other health professions.

## 2. Project description

### 2.1. Concept development

Kern’s model for curriculum development was used in the preparation of a seminar concept. This established method has proved its effectiveness and has been used in the health care systems of numerous countries for two decades. With the help of the Kern model, curriculum development, maintenance and improvement can be implemented in a structured manner [15].

The individual development steps are presented below.

The concept was developed for the Hessian Ministry for Social Affairs and Integration (HMSI) by an interprofessional team of two physicians undergoing specialty training in family medicine, a research assistant with a background in physiotherapy, an educational scientist, a pharmacist, a research employee with a healthcare assistant background, a specialist in family medicine, and experienced specialty training instructors.

#### Step 1: Problem identification and general needs analysis

First of all, a survey of existing interprofessional teaching projects (ITP) was conducted on the basis of a literature search. Projects for the promotion of ITP among students, such as the projects supported by the Robert Bosch foundation were the first to be identified [10]. We then checked whether it would be possible to transfer the individual projects to the target group of seminar participants, including seminar participants that had completed their studies or their professional training [16].

##### Step 2: Special needs analysis

In order to achieve appropriate access to the various professions and to ensure that they all began on an equal footing, interviews were conducted with two participants in existing ITP projects. Furthermore, information was shared and networking carried out with other instructors in the field under the umbrella of the Consortium of Institutes of Higher Education in Health and Rehabilitation in Europe (Cohehre) [17], [18]. The results showed that participants in ITP events often had difficulties finding common ground when specific medical issues were at stake (because of different knowledge levels, for example). At the same time, becoming familiar with other professional groups and sharing information with them was considered to be a very rewarding experience. Working together on specific cases was regarded as particularly helpful. We used these approaches in our seminar and derived specific learning objectives accordingly. 

##### Step 3: Goal definition, learning objectives and learning content

The content of the preceding steps was then appraised, resulting in the following overlapping objectives, which reflect the principles of standard international ITP. 

These consist of:

- learning with one another: jointly develop and expand learning content,
- learning from one another: communicate one’s own professional competencies,
- learning about one another: gain knowledge of other professional groups [19].

In order to create a basis, we wanted our seminar to achieve overarching learning objectives that

1. improve communication between individual professions and
2. break down role expectations and stereotypes of individual professions.

##### Step 4: Didactical implementation

One specific task was to try viewing patient-centered treatment objectives from the point of view of different professions within the interprofessional team, and jointly to develop and discuss these objectives. Based on the described analysis of differing ideas of ITP, and on interviews with experts, the implementation of specific learning objectives for the planned seminar were developed in a multi-stage process. 

Furthermore, the development team was interested from the beginning in providing the various professions with the opportunity to become acquainted with one another outside what are generally hierarchical settings at work. The focus thereby was on finding out more about the competencies and range of tasks performed by the other professions, and strengthening fundamental appreciation of one another. 

In a second step, the participants were asked to discuss the obstacles (e.g. lack of time) and possible solutions (e.g. interprofessional team meetings) that in their view affect communication with other professions. Key questions were: “What do I expect of other professions?” and “How can I contribute towards improving communication and collaboration with other professions?” In order to avoid taking a purely theoretical view, it was important to us that a large part of the seminar was spent conducting practical exercises, where possible with a (simulation) patient [20]. Case examples that were included in the seminars were primarily aimed at undertaking interprofessional collaboration and not at learning special competencies associated with a specific disease. In the development of the seminar, particular importance was attached to ensuring that participants not “only” gained experience but also learned practical tools for use in practice. 

We chose to involve seminar instructors from multiple professions and tried to ensure that all participating professions were represented. The seminar instructors rather adopted the role of observers, learning guides and moderators [21]. The implementation methods that were used were based both on international recommendations on setting up a teaching module for communication skills [20], [22], and recommendations stemming from ITP teaching formats [17], [18], [19]. Important learning mechanisms were seen in experience, reflection and dialog [23]. In this regard, the didactical approach to cooperative learning was especially suitable for such heterogeneous groups [24]. Throughout the entire seminar, stimulating and reflection-inciting methods alternated with plenary and small-group sessions [22]. The following course concept was derived from this approach [10], [25].

Based on eight teaching units à 45 minutes, five key topics were defined. The seminar program, along with the various learning units, learning objectives and employed didactical methods are presented in table 1 (Tab. 1) [14]. 

In the morning, the focus was on getting to know the five unfamiliar professions, both personally and from a professional perspective. To achieve this, and after briefly introducing themselves, the participants were first divided into single-profession groups in which they prepared poster walks. They then presented first themselves as individuals and then their professional responsibilities and the everyday challenges they faced. At this point, there was time for questions and discussion. Afterwards, contributions could be made via structured communication channels (based on the SBAR tool) [8]. After the lunch break, a simulation patient represented the case of a geriatric multimorbid patient following discharge from hospital after an acute fall (femoral neck fracture). In a subsequent fishbowl discussion [26], the participants took the patient’s medical history from an interprofessional perspective. For this purpose, the participants compiled their patient-centered treatment objectives and derived cooperation possibilities from them. They then developed treatment plans (medication plan, prescriptions, short handovers between professions) in heterogeneous (cross-profession) groups and used role play to demonstrate them. At the end, all the challenges and difficulties that the participants described when they presented themselves to the group were reintroduced and analyzed with an aim to finding solutions. 

Overall, all the elements of the seminar were structured interactively, using a wide range of methodologies. 

##### Step 5: Implementation

The implementation was part of the existing seminar program for physicians undergoing specialty training at the Competence Center for General Practice in the German state of Hesse. Up to now, the seminars have taken place at the Sankt-Elisabethen Hospital in Frankfurt am Main and at the Institute of General Practice at Goethe University, Frankfurt am Main, further seminars are planned. Thanks to the support of the HMSI, it was possible to provide the seminars at no cost to participants. 

In the seminars, ten places are available for physicians undergoing specialty training and five each for another three professional groups. As the three further professions are required to have as many points of contact with outpatient health care as possible, the team decided to begin by working with physiotherapists, pharmacists and either health care assistants (HCAs), specially qualified medical assistants, or non-physician practice assistants. Representatives of the various professions are recruited through multiple channels (associations, pharmacist societies, alumni-networks, etc.).

##### Step 6: Evaluation

As part of the seminar program, a detailed questionnaire was distributed for evaluation purposes. The questionnaire was adapted according to the specific seminar and included additional pre- and post-test surveys, and the validated, international evaluation questionnaire UWE-IP [27]. 

The evaluation sheet for the seminar program for physicians in specialist training organized by the Hesse Competence Center was based on evaluation sheet recommendations made by the Specialist Training Department of the German College of General Practitioners and Family Physicians (DEGAM). It was therefore possible to compare evaluation results with those of other program seminars. The evaluation sheet, which consists of five open and 20 closed questions, was divided into two parts and contained additional self-assessment questions. Before the event began, participants filled in the first part, which consisted of demographic questions and three self-assessment questions relating to knowledge of the role played by the different healthcare professions, communication and interprofessional collaboration. Responses to the questions were given on a six-point Likert scale with defined endpoints (1=“very good”, 6=“not at all”). The second part was filled in after the seminar was over and consisted of an evaluation of the entire seminar and a further final self-assessment, based on the questions and response categories described above. 

As its psychometric data show good results, the interprofessional questionnaire UWE-IP, which was translated into German by the University of Heidelberg, is recommended for use in the evaluation of interprofessional entities. 

The number of available evaluation sheets is not yet sufficient for a detailed analysis, which we plan after completion of at least a further three seminars. It may then also be possible to assess effectiveness in terms of sustainability in particular. One limitation is, however, that the questionnaire has been very specifically designed for ITP in a university education setting, as this means it is only possible to draw conclusions on ITP in the context of specialty training and for a working population to a limited degree. Over the long term, the UWE-IP will nevertheless be able to provide a detailed picture of different professional groups, as it covers such topics as “communication and teamwork”, “interprofessional interactions” and “interprofessional relationships” [28]. 

The statistical analysis was mostly descriptive (mean, minimum, maximum and standard deviation). We analyzed the participants’ demographic data and their assessment of the entire seminar, as well as differences in their self-assessment before and after completion of the seminar (Wilcoxon-W-Test). The questionnaires were analyzed using the statistical software IBM^®^ SPSS Statistics^®^ (Version 20).

## 3. Results

Overall, 29 persons took part in the first two seminars, of whom 11 were physicians in specialty training, six were physiotherapists, six pharmacists and six HCAs. Ninety percent of participants were women (N=26). The mean age of the participants was 40.0 years, whereby the physiotherapists (mean 43 years) and the HCAs (mean 42 years) were somewhat older than the physicians in specialty training (mean 37.8 years) and the pharmacists (mean 34.5 years). Most of the participants worked in a practice (see table 2 (Tab. 2)).

Ninety-seven percent of the evaluation sheets were returned (N=28). Overall, the seminar was rated very highly (average grade: 1.39; Min: 1; Max: 2; SD: 0.49). A detailed look at the results revealed that the seminar design (average grade:1.32; Min: 1; Max: 2; SD: 0.32), relevance and practical orientation (average grade: 1.39; Min: 1; Max: 4; SD: 0.74) and the seminar’s contribution towards improving one’s own ability to collaborate with other professionals (average grade: 1.18; Min: 1; Max: 2; SD: 0.39), were rated very positively. Particularly the opportunity for personal discussion/networking, becoming acquainted with other professional groups and the presentation of simple communication possibilities such as the SBAR model were highlighted positively in the free-text fields. The participants would like future interprofessional seminars to involve further professional groups, with psychotherapists (N=14), nursing occupations (n=10), occupational therapists (N=8) and speech therapists (N=3) being mentioned most frequently (multiple answers possible). 

A pre-post comparison of the self-assessment of ability to collaborate interprofessionally showed significant improvement (see table 3 (Tab. 3)). Overall, 89.3% of participants considered that joint training courses with other health professions can improve health care. 

## 4. Discussion

### 4.1. Summary of results in the context of the literature

This paper describes the development, content and evaluation of an innovative, interprofessional seminar. After the seminar, participants considered themselves better prepared for interprofessional collaboration, and reckoned their communication skills and their understanding of other health professions had improved. 

The aim of interprofessional training is to help different health professionals develop or improve their understanding of the roles, tasks and competencies of other health professions. As a result, it may be possible to improve the quality of patient health care and the working relationships between different professional groups [29]. It is well known that hierarchical thinking in health care (for which physicians are mostly responsible) and disrespect for other professions can lead to tensions and difficulties in interprofessional collaboration [30]. When designing the seminar, importance was therefore attached to providing ample opportunity for participants to interact and discuss matters with one another. The aim was also to communicate relevant content from the everyday working lives of the individual professions, whether related to medicine or organizational matters. It is well known that “immersing“ oneself in professions other than one’s own leads to greater understanding and appreciation of the competencies of the other professional groups [31]. In our seminar, the use of simulation patients permitted participants to experience “real-life” case scenarios first hand. Furthermore, the simulation patients enabled participants to train specific communication skills [32]. The purpose of the seminar’s focus on communication skills and teamwork in small groups was to allow participants to interact with one another, exchange views and to learn from one another. In addition, use of a shared language supports the error-free transfer of important information [8].

#### 4.2. Participants

The high proportion of female participants is worth mentioning. For physicians undergoing specialty training, these numbers correspond with the regular demographic distribution of participants in our seminars at the Competence Center for Specialist Training in Hesse. In the other participating professions, the distribution of sexes was also fairly standard [33]. The average age reflected the heterogeneity of participants with much and little professional experience. The fact that the topic of interprofessional collaboration is considered important regardless of age may be an indication that the ability to communicate effectively on an interprofessional level is not associated with greater professional experience. This supports the view that it is ideal to provide such a seminar as part of specialty training for physicians and thus to support communication when physicians are beginning their careers. 

#### 4.3. Recruitment

The recruitment of different professional groups can be difficult to organize and extremely time-consuming. Whereas physicians in specialty training can attend the seminars as part of their training to become family practitioners, the other participants must be intrinsically motivated to take part. If their employer does not support participation in the seminar, it is necessary for those that take part to “sacrifice” their vacation days or, if they are self-employed, to accept a possible shortfall in revenues. This makes it particularly difficult to recruit physiotherapists. It is therefore necessary to reduce obstacles to the participation of this professional group in the seminar. One possibility would be to cooperate with universities and to offer the seminar as an elective in the curriculum of a Master’s degree in physiotherapy, therapy sciences etc. Furthermore, such seminars could be attended in the evenings or at weekends, whereby the need to forego leisure time might again limit interest in taking up such an offer. Up to now, we were able to offer the incentive of eight continuing education credits (issued by the German Physiotherapy Association) for one seminar day. 

#### 4.4. Evaluation

The very good evaluation results reflect a successful and needs-oriented seminar design. The significant improvement shown in the pre-post self-assessment of ability to collaborate interprofessionally could also be followed up after a few weeks or months in order to evaluate the development more precisely and to investigate the influence of the seminar over the long term (see outlook). As participation in the seminar was voluntary, we assume that the interest of the participants in interprofessional collaboration was higher than average. Furthermore, it is impossible to draw general conclusions because of the relatively low number of 29 participants.

## 5. Conclusion

Overall, the evaluation of the seminar was very positive. In the free text fields, participants expressed an interest in the integration of further professional groups (psychotherapists, nursing services, occupational therapists and speech therapists) into a similar seminar format. In response to this wish, psychotherapists will be included in coming seminars. The inclusion of participants that are based locally is also an interesting idea and one that will, if possible, be considered in the planning of seminars in the future. 

We also plan to extend the evaluation to include a follow-up survey after six and 12 months. The results could contribute towards further improvement in the design and content of the seminar, as well as differentiation in the needs of the various professions in accordance with their employment situation (e.g. in inpatient or in outpatient care).

Even if the small number of participants and the fact that the questionnaire was self-developed mean the results of this investigation only serve to indicate a trend and cannot be generally extrapolated to include further seminars, we would like to use the positive results to stress the usefulness of interprofessional seminars and encourage colleagues to establish similar courses as part of specialty training. After completing further seminars, a further publication is also planned, which will include a larger number of respondents, as well as explorative analyses of sub-groups.

## Authorship

The first authorship is shared by Katharina Dippell and Maria-Sophie Brueckle. 

## Acknowledgements

We would like to thank the Hessian Ministry for Social Affairs and Integration (HMSI) for the financial support without which the seminars could not have been provided to participants free of charge. Special thanks also go to Mrs. Andrea Schlicker and Prof. Dr. Mirjam Körner for supporting us with their experience of ITP events, and to the Department of General Practice and Health Services Research at the Heidelberg University Hospital (Germany) for the permission to use their translation of the UWE-IP for our seminar. Further cordial thanks go to the Hesse Chamber of Pharmacists, the Central Association of Physiotherapists, the Hesse Association of General Practitioners and the participating teaching practices of Goethe University Frankfurt am Main. Thank you to Mrs. PD Riphaus for providing facilities at St. Elisabethen Hospital. We would also like to thank all simulation patients for their acting performance and the simulation patient center of Faculty 16 of Goethe University Frankfurt am Main for preparatory training. Our special thanks also go to Phillip Elliot from Goethe University for translating our manuscript to English. And finally, we would like to express our gratitude to all participants in the seminars on interprofessional collaboration. 

## Competing interests

The authors declare that they have no competing interests. 

## Figures and Tables

**Table 1:**
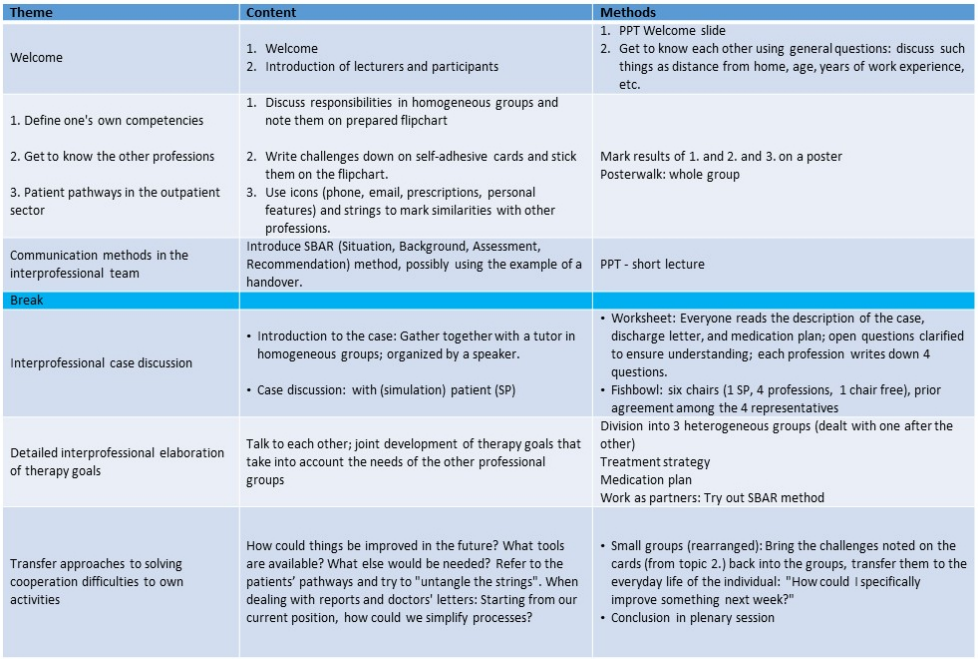
Seminar schedule

**Table 2:**
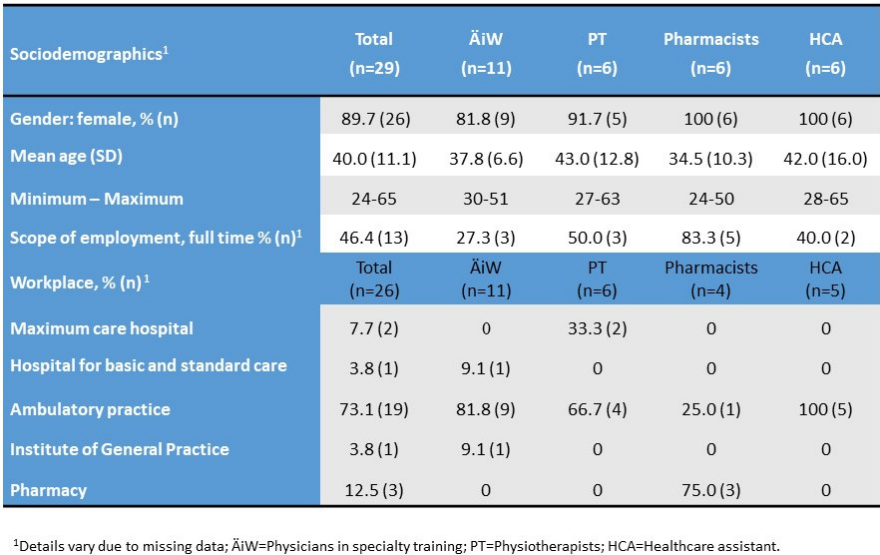
Sociodemographic data of seminar participants

**Table 3:**
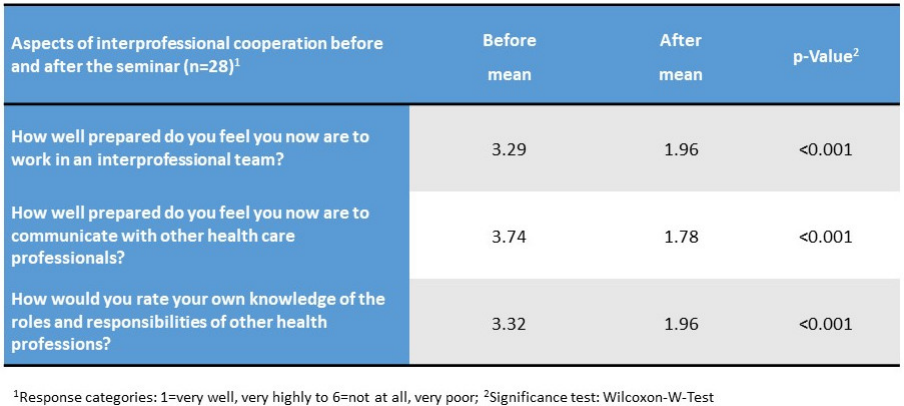
Self-assessment on aspects of interprofessional collaboration before and after seminar participation.
